# Clinical and Magnetic Resonance Imaging Characteristics of Pediatric Acute Disseminating Encephalomyelitis With and Without Antibodies to Myelin Oligodendrocyte Glycoprotein

**DOI:** 10.3389/fped.2022.859932

**Published:** 2022-05-20

**Authors:** Meifang Lei, Yaqiong Cui, Zhaoying Dong, Xiufang Zhi, Jianbo Shu, Chunquan Cai, Dong Li

**Affiliations:** ^1^Department of Neurology, Tianjin Children’s Hospital (Tianjin University Children’s Hospital), Tianjin, China; ^2^Tianjin Pediatric Research Institute, Tianjin Children’s Hospital (Tianjin University Children’s Hospital), Tianjin, China; ^3^Tianjin Key Laboratory of Birth Defects for Prevention and Treatment, Tianjin, China; ^4^Department of Neurology, Tianjin People’s Hospital, Tianjin, China

**Keywords:** acquired demyelinating syndromes (ADSs), acute disseminated encephalomyelitis (ADEM), myelin oligodendrocyte glycoprotein-IgG (MOG-IgG), pediatric, magnetic resonance imaging (MRI)

## Abstract

**Background:**

Myelin oligodendrocyte glycoprotein-immunoglobulin G (MOG-IgG)-associated disorders (MOGADs) have been considered as a new inflammatory disease entity of the central nervous system (CNS) and have heterogeneous clinical and imaging presentations. Acute disseminated encephalomyelitis (ADEM) is one of the most important phenotypes. Our research is aimed to compare the clinical and magnetic resonance imaging (MRI) characteristics of ADEM with or without MOG-IgG in pediatric-acquired demyelinating syndromes (ADSs).

**Methods and Results:**

We retrospectively reviewed the clinical characteristics, MRI features, and outcomes of pediatric patients with ADSs from March 2017 to February 2021 in our center. MOG-IgG was analyzed by transfected cell-based assay (CBA). Among 46 children with ADEM, 21 children (11 girls and 10 boys) were positive for MOG-IgG. Headache, fever, vomiting, vertigo, ataxia, and decreased muscle strength were common in all enrolled children. No significant difference existed in demographic characteristics, symptoms at an initial episode, or laboratory cerebrospinal fluid (CSF) findings between children with MOG-IgG and children without MOG-IgG. For children with MOG-IgG seropositive ADEM, cerebral MRI showed widespread, poorly demarcated bilateral lesions, especially in cortical and subcortical white matter, and spinal MRI often showed lesions spanning more than three segments. The significant difference in MRI features between the two groups was the presence of lesions in the thalamus and cortical area (*p* < 0.05). Most children in both groups showed clinical improvement 1 week after immunotherapy and achieved recovery during their hospital stay. Three children with MOG-IgG and four children without MOG-IgG had one or more relapsing courses with median interattack intervals of 4 (range: 1–7) months and 10 (range: 1–24) months, respectively. New clinical symptoms and lesions on cerebral and spinal MRI were found during relapsing courses in two groups. No recurrences were recorded 6–51 months after each patient’s last episode.

**Conclusions:**

There was no significant difference in clinical characteristics between ADEM children with MOG-IgG and ADEM children without MOG-IgG. For children with MOG-IgG seropositive ADEM, cerebral MRI showed large, bilateral lesions and spinal MRI often showed lesions spanning more than three segments. Children achieved a favorable outcome regardless of MOG-IgG serostatus.

## Introduction

Acute disseminated encephalomyelitis (ADEM) is an inflammatory demyelinating disease of the central nervous system (CNS) that is typically characterized by encephalopathy with polyfocal clinical symptoms ranging from behavioral changes to alterations in consciousness. Magnetic resonance imaging (MRI) in patients with ADEM shows diffuse, poorly demarcated, large (>1–2 cm) lesions predominantly in the white matter and spinal cord (1). The clinical features of ADEM typically follow a monophasic disease course with a favorable prognosis.

Myelin oligodendrocyte glycoprotein (MOG) is a glycoprotein that is exclusively expressed on the surface of myelin sheaths and oligodendrocytes (2, 3). Available research and review have now established a possible role for MOG-immunoglobulin G (IgG) that was associated with a very heterogeneous age-dependent clinical presentation and multiple sclerosis (MS)-typical demyelination and oligodendrocyte pathology, defined as Myelin oligodendrocyte glycoprotein-immunoglobulin G (MOG-IgG)-associated disorders (MOGADs) (4, 5). In recent years, MOGAD has been proposed and considered a new inflammatory disease entity of the CNS (6, 7). The main clinical phenotypes of MOG-IgG-associated demyelinating syndromes change with age, as ADEM-like presentations [ADEM, ADEM-optic neuritis (ON), and multiphasic ADEM] are more common in children <9 years old, and optical-spinal presentations (ON and myelitis) are more common in children aged >9 years and adults (8, 9). Previous studies have indicated that approximately 40% of children with ADEM and nearly 100% of children with multiphasic disseminated encephalomyelitis (MDEM) were seropositive for MOG-IgG (10). Previous studies have reported the clinical and neuroradiological characteristics of pediatric ADEM with MOG-IgG (11–14). The clinical and MRI characteristics of patients with ADEM with or without MOG-IgG in northern China have not been reported.

In this work, we retrospectively delineated the demographic and clinical characteristics, laboratory tests of cerebrospinal fluid (CSF), and MRI features of patients with pediatric ADEM with or without MOG-IgG from northern China.

## Materials and Methods

### Patients

Patients with acquired demyelinating syndromes (ADSs) and onset age prior to 18 years were enrolled in this study. The criteria for diagnosing pediatric ADEM followed the International Pediatric Multiple Sclerosis Study Group (IPMSSG) criteria (1). Children were diagnosed with ADEM according to the following criteria: (a) a first polyfocal, clinical event with presumed inflammatory demyelinating causes; (b) encephalopathy that could not be explained by fever; (c) no new clinical and MRI findings emerging 3 months or more after the onset; and (d) typically abnormal brain MRI findings during the acute phase.

**FIGURE 1:**
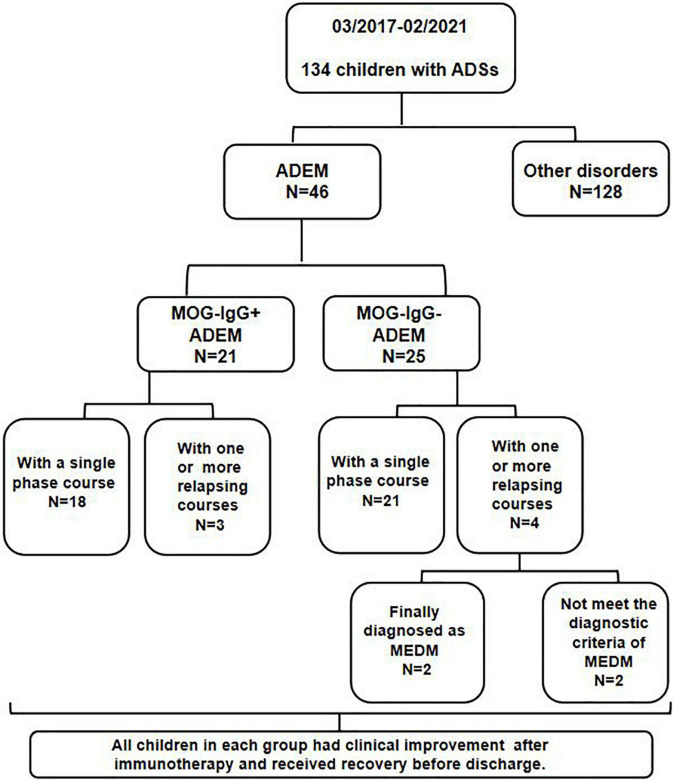
Flowchart of samples and results.

All children were from Tianjin Children’s Hospital from March 2017 to February 2021. A serum MOG-IgG test was performed on all enrolled children by transfected cell-based assay (CBA). The flowchart of patients and results is shown in Figure 1.

### Clinical Data

Clinical symptoms and signs were classified as headache, fever, vomiting or vertigo, aphasia or dysarthria, ataxia, sensory symptoms, visual disturbance, epileptic seizure, nuchal rigidity, decreased muscle strength, spasticity, and sphincteric dysfunction. Lumbar puncture was performed on all enrolled children at disease onset. cerebrospinal fluid (CSF) samples were collected. CSF analysis included the white blood cell (WBC) count, the total protein level, and oligoclonal bands (OCBs).

### Magnetic Resonance Imaging Analysis

Magnetic resonance imaging of the brain and spinal cord was performed by a Siemens Avanto 1.5T MRI unit (Siemens, Germany), and MRI results were evaluated by a pediatric neurologist and a radiologist who were blinded to the clinical diagnosis. MRI scans included T1-axial, T2-axial, T2-sagittal, fluid-attenuated inversion recovery (FLAIR)-axial, and diffusion-weighted imaging (DWI). The lesion location of the brain and spinal cord was classified as white matter (such as subcortical and paraventricular), corpus callosum, optic nerve, thalamus, basal ganglia, cerebellum, brainstem, cortical area, and spinal cord. Typical cerebral and spinal cord lesions were defined according to the previous reports (15, 16).

### Statistical Analysis

Statistical analysis was performed by IBM SPSS, release v.24.0 (IBM Corporation). Continuous variables between groups were compared by the Mann–Whitney test. Categorical variables were compared by the chi-square (χ^2^) test or Fisher’s exact test. Statistical significance was defined as a 2-sided *p* < 0.05.

## Results

### Clinical Characteristics

Of the 134 children with ADSs, 46 children (21 girls and 25 boys) were finally diagnosed with ADEM. Among these patients, 21 children (11 girls and 10 boys) were positive for MOG-IgG. They had a median follow-up of 23 (range 2–39) months. The demographic and clinical features of the enrolled children are shown in Table 1. Statistical differences were calculated between children in the ADEM with MOG-IgG group and children in the ADEM without MOG-IgG group. There was no significant difference between the two groups in age at symptom onset, sex, or symptoms at an initial episode. We found that the percentage of patients with a preceding infection was lower in children with MOG-IgG seropositive ADEM than in children with MOG-IgG seronegative ADEM (*p* = 0.023).

**TABLE 1:** Demographic, clinical, and cerebrospinal fluid (CSF) features of ADEM children with or without myelin oligodendrocyte glycoprotein-immunoglobulin G (MOG-IgG).

Patients	MOG-IgG + ADEM (*N* = 21)	MOG-IgG- ADEM (*N* = 25)	*x* ^2^ */Z*	*P*
Female, *N* (%)	11 (52.4%)	10 (40.0%)	0.705	0.401^#^
Age at onset (years)	7.0 (3.0–8.0)	6.0 (4.0–7.0)	−1.899	0.059
**Symptoms at initial episode**				
Headache	6 (28.6%)	9 (36.0%)	0.287	0.592^#^
Fever	12 (57.1%)	14 (56.0%)	0.006	0.938^#^
Vomiting or vertigo	12 (57.1%)	12 (48.0%)	0.382	0.536^#^
Aphasia or dysarthria	5 (23.8%)	5 (20.0%)	0.000	1.000^#^
Ataxia	12 (40%)	8 (32.0%)	0.456	0.087^#^
Sensory symptoms	1 (4.8%)	1 (4.0%)	–	1.000^§^
Visual disturbance	8 (38.1%)	8 (32.0%)	0.187	0.665^#^
Epileptic seizure	5 (23.8%)	8 (32.0%)	1.843	0.539^#^
Nuchal rigidity	7 (33.3%)	10 (40.0%)	1.885	0.641^#^
Decreased muscle strength	9 (42.9%)	13 (52.0%)	3.069	0.536^#^
Spasticity	5 (23.8%)	5 (20.0%)	0.000	1.000^#^
Sphincteric dysfunction	2 (9.5%)	5 (20.0%)	0.329	0.428^#^
Preceding infection	6 (28.6%)	16 (64.0%)	5.741	**0.017** ^#^ *
Readmission	3 (14.3%)	4 (16.0%)	0.063	0.802^#^

*ADEM: acute disseminated encephalomyelitis.*

*^#^Chi square test.*

*^§^ Fisher’s exact test.*

*With statistical significance.

### Cerebrospinal Fluid Studies

The CSF results are detailed in Table 2. No significant difference existed between the two groups. We found that the level of CSF protein was increased and OCBs rarely appeared in most of the enrolled children (refer to Table 3). Autoantibodies, such as anti-AQP4 IgG and anti-NMDA receptor antibodies, were negative in all enrolled patients. All the enrolled children were negative for etiological detection, which included Epstein-Barr virus (EBV), tuberculosis (TB), mononuclear phagocytes (MP), and herpes simplex virus (HSV)-DNA (data not shown).

**TABLE 2:** Laboratory values of cerebrospinal fluid (CSF) in ADEM children with or without myelin oligodendrocyte glycoprotein-immunoglobulin G (MOG-IgG).

	MOG-IgG + ADEM (*N* = 21)	MOG-IgG- ADEM (*N* = 25)	*x* ^2^	*P*
CSF elevated white blood cell count, >5 cells/μl	14 (66.7%)	17 (68.0%)	0.009	0.923^#^
Elevated CSF protein, >50 mg/dL	19 (90.5%)	23 (92.0%)	0	1.000^#^
oligoclonal bands (OCBs)	2 (9.5%)	3 (12.0%)	0	1.000^#^

*CSF, cerebrospinal fluid; ADEM, acute disseminated encephalomyelitis. ^#^Chi square test.*

**TABLE 3:** Presence of the lesion in specific areas of the brain and spinal cord.

	MOG-IgG + ADEM (*N* = 21)	MOG-IgG- ADEM (*N* = 25)	*x* ^2^	*P*
**White matter**				
Subcortical	11 (52.4%)	16 (64.0%)	0.636	0.425^#^
Paraventricular	2 (9.5%)	2 (8.0%)	0.000	1.000^#^
Corpus callosum	0 (0%)	4 (16.0%)	1.941	0.114^#^
Optic nerve	2 (9.5%)	0 (0%)	–	0.203^§^
Thalamus	12 (57.1%)	6 (24.0%)	5.263	**0.022** ^#^ *
Basal ganglia	8 (38.1%)	9 (36.0%)	0.022	0.883^#^
Cerebellum	7 (33.3%)	9 (36.0%)	0.036	0.850^#^
Brainstem	8 (38.1%)	6 (24.0%)	1.071	0.301^#^
Cortical area	15 (71.4%)	9 (36.0%)	5.741	**0.012** ^#^ *
Spinal cord	9 (42.9%)	15 (60.0%)	1.344	0.246^#^

*ADEM: acute disseminated encephalomyelitis.*

*^#^Chi square test.*

*^§^ Fisher’s exact test.*

*With statistical significance.

### MRI Finding

**FIGURE 2:**
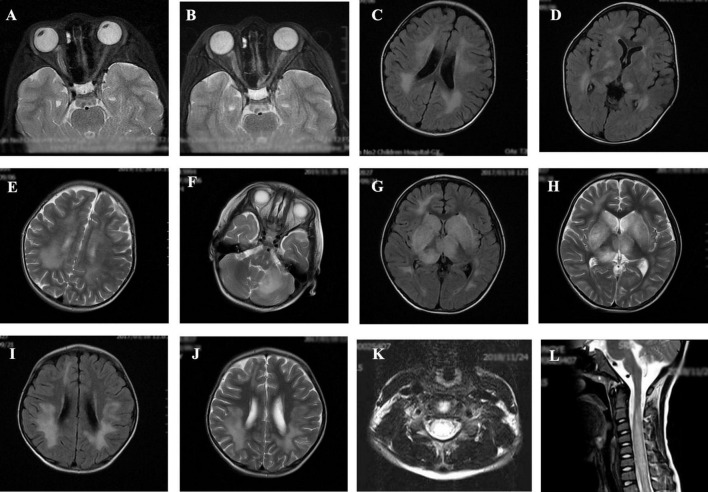
Magnetic resonance imaging (MRI) results of acute disseminated encephalomyelitis (ADEM) children with or without myelin oligodendrocyte glycoprotein-immunoglobulin G (MOG-IgG. **(A,B)** (T2-axial) cerebral MRI of a 7-year-old boy with MOG-IgG seropositive ADEM showed small and blurred lesions of optical nerve. **(C–F)** [T2-axial, fluid-attenuated inversion recovery (FLAIR)-axial] cerebral MRI of a 2-year-old girl with MOG-IgG showed large, blurred lesions in bilateral white matter areas of frontal, parietal, and temporal lobes, bilateral basal ganglia, bilateral thalamus, and left cerebellar hemisphere. **(G,H)** (T2-axial, FLAIR-axial) cerebral MRI of a 4-year-old boy without MOG-IgG revealed a lesion in bilateral basal ganglia, thalamus, periventricular white matter, and right frontal lobe. **(I,J)** (T2-axial, FLAIR-axial) cerebral MRI of a 4-year-old girl with MOG-IgG seronegative ADEM showed large and blurred lesions in bilateral white matter areas of the frontal parietal. **(K,L)** (Sagittal-T2) Spinal MRI of a 2-year-old girl with MOG-IgG revealed prominent involvement of cervical and thoracic spinal lesion (≥3 segments).

The MRI characteristics of the brain and spinal cord imaging are summarized in Table 3 and typical MRI scans of five children are shown in Figure 2. Children with MOG-IgG seropositive ADEM presented with more lesions in the thalamus and cortical area than children with MOG-IgG seronegative ADEM, and this difference was significant (*p* < 0.05).

### Treatment

All 46 children with ADEM received high-dose glucocorticoid therapy within 15 days of the first acute attack. Children who were positive for MOG-IgG were treated with intravenous methylprednisolone, followed by oral methylprednisolone. Fourteen (93.33%) children were also treated with intravenous immunoglobulins (IVIG). Among the 25 children negative for MOG-IgG, twenty-three (92.0%) children were treated with intravenous methylprednisolone and IVIG, followed by oral methylprednisolone. Two (8.0%) children received intravenous dexamethasone and IVIG, followed by oral methylprednisolone. The median time from initial treatment to an improvement in clinical symptoms was 6 (range: 2–41) days for children with MOG-IgG seropositive ADEM and 7 (from 1 to 42) days for children with MOG-IgG seronegative ADEM (*p* = 0.88). Most of the enrolled children (39/46) achieved clinical recovery during their hospital stay.

### Relapse

For the 21 children with MOG-IgG seropositive ADEM, three children had one or more relapsing courses. One patient had a total of 4 ADEM episodes and the other two children had 2 ADEM episodes. The median interattack interval was 4 (range: 1–7) months. During relapsing courses, the clinical symptoms included fever, vomiting or vertigo, visual disturbance, and epileptic seizure, which were all present during the onset attack. We also found new MRI lesions on the cerebral and spinal cord when compared with the first episode. Among the 25 children in the ADEM without MOG-IgG group, two children had 2 ADEM episodes, one patient had 3 ADEM episodes and one patient had 4 ADEM episodes. The median interattack interval was 10 (range: 1–24) months. Both new symptoms and new MRI lesions of the cerebral and spinal cord were found in only two children during the relapsing courses, and the two patients were finally diagnosed with MDEM. After immunotherapy, all children with relapsing courses in each group achieved clinical improvement and recovery before discharge. At the last follow-up, 6–51 months after the last episode, no recurrence was recorded in any enrolled patient.

## Discussion

In recent years, MOG-IgG-associated demyelination syndrome has been extensively studied (9, 17–19). A large prospective cohort study indicated that MOG-IgG existed in one-third of children during demyelinating attacks. Ninety-six percent of MOG-IgG-positive children presented with a combination of ADEM, ON, and transverse myelitis (TM) (20). Most MOG-IgG-positive children experience a monophasic disease. Clinical relapses were more common in children with persistent seropositivity, although not exclusively (21).

In our research, we compared the demographic and clinical characteristics and MRI features between ADEM children with MOG-IgG and ADEM children without MOG-IgG, which was based on the updated ADEM guidelines by the IPMSSG (1).

Of all 134 children with ADSs, 46 children were diagnosed with ADEM. Among the 46 children with ADEM, 21 children were positive for MOG-IgG. For children with MOG-IgG seropositive ADEM, the median age at disease onset was 7.0 (from 3.0 to 8.0) years and the ratio of men to women was 1.0:1.0. The results were in accordance with a previous report, in which the age at clinical onset was 7.31 with a range from 4.93 to 10.57 and the female percentage was 50% (20). Regarding clinical characteristics, 40% of children with MOG-IgG seropositive ADEM were presented with ataxia, while 32% of children with MOG-IgG seronegative ADEM had ataxia; the difference was not statistically significant. This finding was not consistent with a cohort study that delineated the clinical data of ADEM with and without MOG-IgG in China. In this study, MOG-IgG seropositive children had significantly more ataxia (*p* = 0.025) (22).

Pleocytosis was recorded in 14 (66.7%) of 30 children with MOG-IgG and 17 (68.00%) of 25 children without MOG-IgG; this difference was not significant. This result indicated that there was an inflammatory burden of the CNS in the acute attack regardless of MOG-IgG (13). This finding was not consistent with a previous report, in which the number of CSF leukocytes was significantly increased in children with MOG-IgG when compared with children without MOG-IgG (13, 23, 24). We also found that children with MOG-IgG (2/21, 9.5%) had a lower OCB rate than children without MOG-IgG (3/25, 12.00%), which was similar to a previous study. In this study, OCBs were rarely present in the CSF of children with MOG-IgG when compared with children without MOG-IgG (23). This phenomenon may contribute to the different pathophysiology of MOG-IgG-associated demyelination syndromes as compared to other demyelination syndromes of the CNS.

Typical MRI lesions were detected in all enrolled children. In both groups, the most common lesion areas were in the subcortical white matter, thalamus, and spinal cord, which was consistent with a previous study (25). Children in the MOG-IgG seropositive ADEM group were more likely to present with large lesions in widespread locations, particularly in the cortical area, subcortical white matter, and thalamus. According to the E.U. pediatric MOG consortium consensus, ADEM is the largest subgroup of pediatric MOGAD (40–50%). The typical MRI shows bilateral supratentorial brain lesions, which are mostly located on the subcortical and deep white matter and the deep gray matter. Lesions are T2-hyperintense, large (>2 cm in size), blurred, and poorly demarcated. For the spinal cord, the lesion is often characterized by extending over three or more vertebral segments and the gray matter is preferentially involved (26). In our study, for children in the MOG-IgG seronegative ADEM, the locations of the lesion were mainly presented in subcortical, cerebellum, basal ganglia, and spinal cord, and the lesion in the spinal cord extended over three segments, which was in accordance with the finding of the above consensus and a previous research (13). Most of the lesions showed large patches of high intensity in T2-weighted imaging (T2WI) and FLAIR sequences, and the boundary of the lesions was not clear. This finding was similar to previous reports (27, 28). The lesion in the optic nerve was present in two out of 21 children with MOG-IgG and zero out of 25 children without MOG-IgG, while the difference between the two groups was not statistically significant. This result was in contrast with that of another study, which showed that children had a similar chance of presenting with lesions in the optic nerve regardless of MOG-IgG status (22).

According to the latest treatment methods for MOG-IgG-associated demyelination in children, first-line immunotherapy conventionally includes intravenous corticosteroids, IVIG, and plasma exchange (PLEX) in a single or combined form (29). Currently, corticosteroids are clinically considered a very useful therapeutic method, as they can help to reduce inflammation, seal the blood-brain barrier, and reduce antibody production (30). MOG-IgG-positive children with a monophasic course often have a favorable prognosis in the context of undetectable MOG antibody levels, similar to children negative for MOG-IgG with a monophasic course (9, 24). In this study, the clinical outcomes of children with or without MOG-IgG were favorable, although some children in each group suffered recurrences. The diagnosis of MDEM revised by IPMSSG was referred for the diagnosis of MDEM (1). Only two children (≥2 ADEM episodes) were finally diagnosed with MDEM. Relapsing courses following ADEM, which occurs beyond a second attack indicates a chronic disorder and is more likely to be diagnosed as MS or neuromyelitis optica (NMO) (31).

The present study has several limitations. First, because of the retrospective design of this study, the clinical symptoms and follow-up were not accurate. Second, owing to the small sample size and the single-center nature of this study, this study sample had some limitations and was not representative of the majority of the pediatric population in northern China. Third, although no recurrence occurred in patients enrolled in the last period of 2020, the follow-up time for these patients was relatively short, which may have interfered with the outcome data. Finally, ADEM is a heterogeneous entity and has been more viewed as a “syndrome” rather than a specific disorder (1). Thus, the comparison between ADEM with or without MOG-IgG was narrow for pediatric ADS of the CNS. In the future studies, comparisons of the clinical characteristics of various demyelinating diseases (e.g., MOGAD, MS, and AQP4-ab-positive NMOSD) in a multicenter cooperation and with a large sample size and long follow-up time are needed.

## Conclusion

In summary, children with MOG-IgG seropositive ADEM had clinical characteristics and MRI features that were different from those of children without MOG-IgG, although this difference was not statistically significant. Cerebral MRI showed large, bilateral lesions in the white matter of cortical and subcortical, and spinal MRI often showed lesions spanning more than three segments. The clinical prognosis was favorable for most children with or without MOG-IgG and children with relapses had no further attacks during the follow-up.

## Data Availability Statement

The raw data supporting the conclusions of this article will be made available by the authors, without undue reservation.

## Ethics Statement

The studies involving human participants were reviewed and approved by the Ethics Committees of Tianjin Children’s Hospital. Written informed consent to participate in this study was provided by the participants’ legal guardian/next of kin.

## Author Contributions

ML, DL, and CC: study idea and design. ML and DL: clinical samples. YC, XZ, and ZD: experiment. YC, XZ, JS, and ZD: data analysis. ML and YC: manuscript. DL and CC: critical review of the manuscript. All authors approval final version.

## Conflict of Interest

The authors declare that the research was conducted in the absence of any commercial or financial relationships that could be construed as a potential conflict of interest.

## Publisher’s Note

All claims expressed in this article are solely those of the authors and do not necessarily represent those of their affiliated organizations, or those of the publisher, the editors and the reviewers. Any product that may be evaluated in this article, or claim that may be made by its manufacturer, is not guaranteed or endorsed by the publisher.

## Abbreviations

ADEM: acute disseminated encephalomyelitis
ADSs: acquired demyelinating syndromes
CBA: cell-based assay
CNS: central nervous system
IVIG: intravenous immunoglobulins
MDEM: multiphasic disseminated encephalomyelitis
MOG: myelin oligodendrocyte glycoprotein
MOGAD: MOG-IgG-associated disorders
MRI: magnetic resonance imaging
MS: multiple sclerosis
NMO: neuromyelitis optica
ON: optic neuritis
TM: transverse myelitis.
